# Feasibility study of an intervention program to enhance self-confidence of kindergarten teachers who deal with radiation-related health concerns from parents with young children

**DOI:** 10.1186/s40814-022-00993-6

**Published:** 2022-02-03

**Authors:** Nobuaki Moriyama, Chihiro Nakayama, Kiyotaka Watanabe, Tomomi Kuga, Seiji Yasumura

**Affiliations:** 1 Department of Public Health, Fukushima Medical University School of Medicine, 1, Hikarigaoka, Fukushima, 960–1295 Japan; 2 Department of Medicine, School of Medicine, Teikyo University, Tokyo, Japan

**Keywords:** Radiation accident, Self-confidence, Literacy, Radiation-related health concerns, Combined intervention

## Abstract

**Background:**

Following the Fukushima Daiichi Nuclear Power Station accident in March 2011, radiation anxiety was high among residents in affected areas. Enhancing radiation-related health literacy is effective in reducing radiation anxiety. This feasibility study aimed to examine a novel intervention program to enhance the self-confidence of kindergarten teachers who deal with radiation-related health concerns from parents in order to determine the feasibility of conducting a future randomized controlled trial.

**Methods:**

Teachers and administrative staff of two private kindergartens in Fukushima City and members of Media Doctor Japan (a research group for enhancing the quality of health news reporting in Japan) were recruited for study participation. Participants were randomly assigned to intervention and control groups. The intervention group participated in the intervention program, comprised of lectures, group discussions, and presentations. The control group received the same written materials used in the intervention program. The primary outcome of this study was feasibility, assessed via four questions concerning program acceptability and described without quantitative analysis. Secondary outcomes were self-confidence concerning parent consultation (1 question, 4-point Likert scale), radiation-related health knowledge (5 question quiz, 1 point for each correct answer, score: 0–5), and health literacy (assessment developed by Ishilawa, et al., score: 1–5) assessed numerically before and after the intervention. Means and standard deviations of outcomes before and after the intervention and their changes in both groups were reported with groups of participants (kindergartens and the Media Doctor Research Japan) reported separately. No quantitative analyses were performed for secondary outcomes.

**Results:**

Five and six kindergarten workers and seven and seven Media Doctor Japan members participated in the intervention and control groups, respectively. Reported acceptability was generally positive, and only one participant gave a negative response regarding program usefulness. Improved self-confidence was found in kindergarten employee participants. Radiation-related health knowledge was higher after the intervention in both kindergarten teachers and Media Doctor Japan members. The amount of change was higher in the intervention group than in the control group.

**Conclusions:**

The intervention program enhanced self-confidence in kindergarten employees. The feasibility of the intervention program for a larger randomized controlled trial was ascertained. Time to conduct lectures and group discussions should be increased to further enhance health literacy.

**Trial registrations:**

UMIN000042527 [University Hospital Medical Information Network (UMIN) Center] registered on November 25, 2020.

## Key messages regarding feasibility

- Participants were recruited for a face-to-face complex intervention program; program effectiveness to enhance kindergarten teachers’ self-confidence in dealing with radiation-related health concerns from parents with young children was uncertain.
- Program participants from the target population reported good acceptability of the intervention program.
- The feasibility of this intervention program was ascertained; subsequent iterations should include a greater number of participants and longer times for lectures and discussions in order to further enhance literacy of participants.

## Background

On March 11, 2011, the Great East Japan Earthquake (GEJE) and subsequent tsunami caused the Tokyo Electric Power Company’s Fukushima Daiichi Nuclear Power Station reactor accident, which resulted in radioactive contamination of the surrounding areas. Although no radiation exposure-related health effects occurred in local residents [1, 2], residents in Fukushima Prefecture experienced extensive lifestyle changes that seemed to have a greater impact than the radiation disaster itself. Thus, the disaster indirectly resulted in various health effects [3, 4], including excess mortality [5] among local older people. Indeed, the GEJE resulted in not only physical health problems indirectly associated with radiation exposure, but also psychological and social effects [6]. In particular, the GEJE and Fukushima disaster created anxiety regarding the physical risks of radiation exposure in the local population [7]. According to previous findings, this anxiety may have disproportionally affected women with young children. Notably, women with young children were recognized as an at-risk group for symptoms of post-traumatic stress disorder following the nuclear accident at Three Mile Island in 1979 [8]. Additionally, following the GEJE nuclear accident, the proportion of positive screens of depressive symptoms in mothers with infants in Fukushima Prefecture was higher than that reported using the same measure in other regions of Japan [9]. Adams [10] suggested that the poorer psychological well-being of mothers of young children following these accidents can be largely explained by their continued concerns about the physical health risks associated with nuclear accidents; following the infamous Chernobyl accident in 1987, the health risks of nuclear accidents, particularly to young children, became evident. Thus, mothers of young children may fear similar outcomes for their children.

The Fukushima Health Management Survey (FHMS) was planned and implemented to monitor the physical and psychological health status of all Fukushima Prefecture residents [11]. Based on data derived from the Pregnancy and Birth Survey of the FHMS that targeted mothers with infants, 2262 of 8196 mothers (28%) were screened positive for depression in 2011 [9]. Accumulating evidence indicates that radiation anxiety is associated with poor mental health outcome [12, 13], especially in mothers of young children [14]; thus, managing anxiety in women with young children is essential to prevent psychological distress.

A possible factor contributing to radiation anxiety among the public is confusion concerning relevant information. The inaccuracy of public information disseminated to citizens in the initial months after the disaster was highly problematic [15]. Previous studies have examined the effect of information consumption on radiation anxiety according to the information source and demonstrated that information spread by rumor or word of mouth was associated with increased health-related fear and anxiety [16, 17]. Indeed, residents of Fukushima Prefecture were confounded by information disseminated by the mass media [18]. Health literacy (HL) is key to avoid confusion cause by such unfamiliar information. HL is defined as the cognitive and social skills that determine the motivation and ability of individuals to gain access to, understand, and use information in ways that promote and maintain good health [19]. Nutbeam [20] classified HL into three categories: functional HL (the ability to obtain relevant health information and apply that knowledge to a limited range of prescribed activities), communicative HL (the ability to extract information and to apply information to changing circumstances), and critical HL (the ability to analyze information critically and use this information to exert greater control over life events and situations). A previous study has shown that high communicative and critical health literacy (CCHL) is associated with low radiation anxiety among Fukushima residents [21], suggesting that improved CCHL is effective in reducing radiation anxiety. Additionally, Nutbeam [22] claimed the importance of prior knowledge with which conceptual model of health literacy commences.

After the nuclear accident in Fukushima, teachers working in kindergartens were frequently consulted by parents with young children about radiation-related health issues [23]. Mothers were most worried about food as a possible source of internal contamination [24]. Small consultation meetings were held in kindergartens by several specialists [25]; however, the effectiveness of these measures in targeting the staff members who usually field these concerns to reduce anxiety and enhance self-confidence has not been reported. Thus, the purpose of this study was to verify the feasibility of a novel intervention program targeting kindergarten teachers in order to enhance participant HL and self-confidence in dealing with radiation-related health concerns received from parents. Accordingly, this study (1) determined the effectiveness of the content and delivery methods used in the intervention program, (2) evaluated the appropriateness of potential outcome measures for future studies, and (3) refined the intervention contents for future randomized controlled trials (RCTs) with larger sample sizes.

## Methods

### Study design

This feasibility study was designed to explore test procedures for acceptability, compliance, delivery of the intervention, recruitment, and retention of an intervention program in accordance with guidelines for developing and evaluating complex interventions by the Medical Research Council [26]. This study was a randomized controlled trial.

### Participants

Participants were recruited from nursery teachers and administrative staff working at two private kindergartens in Fukushima City. In addition, participants were also recruited from Media Doctor Japan, a research group chaired by Dr. Kiyotaka Watanabe (co-author of this paper). The Media Doctor Japan group has been researching the state of media coverage of medical research news reported through bi-monthly meetings, and members include medical professionals, journalists, librarians, patients, and others. Media Doctor Japan members were included in this feasibility study based on their extensive experiences with critical appraisal of health news reported by Japanese newspapers, websites, and TV programs, with the expectation that they could provide helpful feedback for improving the intervention program for future trials. After ethics review and approval, we recruited participants for this study from two kindergartens by asking a representative from each kindergarten to encourage staff to participate in this study. Media Doctor Japan participants who were part of the Media Doctor Japan mailing list were recruited via e-mail.

A total of 12 kindergarten teachers and administrative staff working at private kindergartens in Fukushima City were randomly assigned to two groups. One group received the face-to-face intervention described below, and the control group received only the materials used in the intervention program. A total of 14 participants from the Media Doctor Japan research group were also randomly assigned to two groups in the same way as the participants in Fukushima City described above. The online intervention program was conducted in the same manner for both groups.

### Sample size

A sample size of 30 was previously planned for this feasibility study according to the suggestion of a previous study [27]. In this study, approximately 10 participants from two kindergartens and approximately 20 participants from the Media Doctor Japan research group were expected.

### Randomization

Participants were randomized into one of two groups using computer-generated random numbers. Effective blinding was not possible because both the participants and researchers clearly understood the differences between the two groups.

### Participant flow

Of the 20 workers employed at the selected kindergartens, 12 (60%) agreed to participate in the study, and 14 out of 19 Media Doctor Japan members (74%) agreed to participate in this study. Of the 12 participating kindergarten employees, six were assigned to the intervention group, and six were assigned to the control group. One participant in the intervention group did not appear in the intervention program, and one participant was replaced by another in the control group because of work commitments. Four out of six (67%) of those originally assigned to the intervention group followed the protocol, and five out of six (83%) in the control group participated in the assigned program. The overall compliance rate was 75% (9/12).

**Fig. 1:**
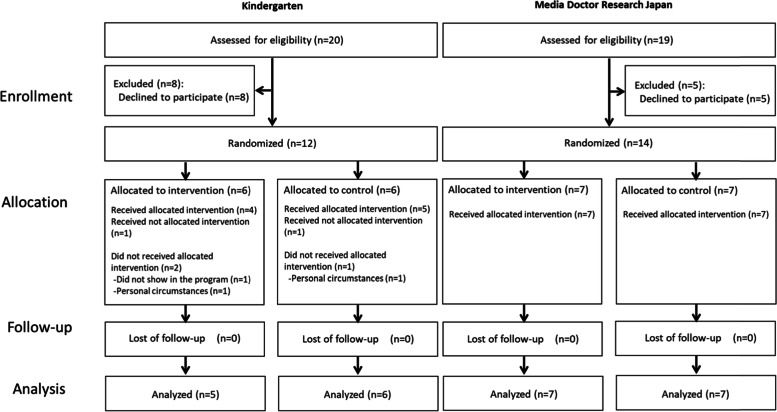
Participant flow

Of the 14 participants from Media Doctor Japan, seven were assigned to the intervention group, and seven were assigned to the control group. One participant in the control group left the online discussion meeting room prior to the conclusion of the program because of Internet connection problems; one participant had difficulty navigating from the URL to the online response form and was not able to respond in real-time (judged as non-compliant in this report). The compliance rates were 100% (7/7) in the intervention group and 71% (5/7) in the control group, and the overall compliance rate was 85% (12/14) (Fig. 1).

### Intervention details

Kindergarten employees were asked to gather at a venue in Fukushima City and participate in the face-to-face intervention program described below. Since participants from the Media Doctor Japan lived far from each other, the intervention was conducted using Zoom, a videoconferencing tool. The intervention program was held during the daytime on weekends.

This intervention was performed once, and the duration was approximately 120 min. The intervention program, which was the same in both the face-to-face and online formats, consisted of (1) introduction (10 min), (2) lecture (50 min), (3) group discussion (40 min), (4) presentation (10 min), and (5) summary (10 min). The introduction aimed to explain the purpose of this intervention program to the target audience. Lectures were divided into two parts: “Radiation and Health Effects” (20 min) and “Media Literacy” (30 min). The lecture on “Radiation and Health Effects” aimed to provide knowledge regarding radiation and its health effects. The topics, which were chosen from what was unclear and inflated public anxiety, included properties of radiation, association of radiation exposure with an incidence of cancers, genetic effects of radiation exposure, mechanism of DNA repair damaged by irradiation, and food reference values. The lecture on “Media Literacy” aimed to enhance participants’ media literacy through demonstration of reading an article from a certain point of view. Group discussions were conducted by dividing the participants into small groups and asking them to share their experiences of dealing with people who asked for advice after the Great East Japan Earthquake and how they dealt with it, as well as what they wished they could have done at that time. Following group discussion, participants were asked to choose a presenter within the group, summarize what was discussed, and give a presentation on this information. This series of activities in this program was designed to enhance health literacy, which consists of various skills.

Approximately 1 week after the intervention group participated in the intervention program, the control group was provided with the same material used in the intervention program to provide equal opportunity for participation in the intervention.

### Data measurement

Following the completion of enrollment, baseline data were collected at a public hall or the researchers’ institution in Fukushima City in January 2021. For participants in the control group, baseline questionnaires were sent and received by mail. Data were assessed using a questionnaire conducted before and after the intervention. Data were obtained from the participants in Fukushima City by asking them to fill out the questionnaire at the venue. For participants in the online intervention group, the URL of the response page created based on the questionnaire was sent via an online meeting system, and participants were asked to enter their responses in the form.

Acceptability was used to assess the feasibility of this intervention program via the following four questions: (1) Do you think the program is useful? (2) Do you think lectures in this program were easy to understand (understandability of lectures), (3) Do you feel the group discussions are easy to participate in (ease of participation in group discussion), and (4) Do you recommend participation in this intervention program to someone you know? (recommendation degree of participation in this program). Participants were asked to respond to questions using a nominal scale, e.g., “strong agree,” “agree,” “neutral,” “disagree,” and “strong disagree.” In addition, participants were asked to describe their impressions of the program in an open-ended format.

Self-confidence in responding to questions about radiation health effects (self-confidence) was considered the primary outcome of future studies with a larger sample size. Possible secondary outcomes included knowledge to cope with questions about radiation health effects (knowledge) and HL. Self-confidence was assessed through the following questions: “Are you confident in your ability to answer questions about radiation and the health effects of radiation?” Participants were asked to respond using a 4-point Likert scale, e.g., “yes,” “rather yes,” “rather no,” and “no.” Responses were scored by assigning 4 points for “yes,” 3 points for “rather yes,” 2 points for “rather no,” and 1 point for “no.”

Participants’ knowledge was assessed by questions about the degree of accurate understanding of the following five areas: properties of radiation, association of radiation exposure with incidence of cancers, genetic effects of radiation exposure, mechanism of DNA repair damaged by irradiation, and food reference values. Participants were asked to answer “true,” “false,” and “not certain” for five short sentences’ questions that were well known and often misunderstood. The correct answer was scored 1 point for each question, and points were summed as knowledge (score: 0–5).

To assess HL, we used the 5-point HL scale developed by Ishikawa et al. [28], which has been used in public. This scale was constructed to measure communicative HL and critical HL. This scale determines whether respondents are able to (1) collect health-related information from various sources, (2) extract their desired information, (3) understand and communicate the obtained information, (4) consider the credibility of the information, and (5) make decisions based on health-related issues. Each item was rated on a 5-point scale ranging from 1 (strongly disagree) to 5 (strongly agree). The individual HL status of each respondent was numerically assessed by obtaining the average scores of all five items.

In addition, demographic characteristics (age, sex, and education), subjective health, mental health, and HL were collected. To assess participants’ mental health, the validated Japanese version of the Kessler 6-item Psychological Distress Scale (K6) [29, 30] was administered. The K6 consists of six brief questions about depression and anxiety symptoms during the past 30 days. All items were measured on a 5-point scale from 0 (never) to 4 (all of the time), and the total score was calculated (range: 0–24), with higher scores representing worse mental health status.

### Data analysis

To determine the effectiveness of the contents and delivery methods for the intervention program, responses to the acceptability of four questions were assessed following the intervention. Descriptive data of each outcome measure (mean ± standard deviation) were summarized to assess the appropriateness of potential outcome measures for a future study. Due to the small sample size in this feasibility study, quantitative statistical analyses of outcome measures and demographic characteristics including age, sex, experience of relocation due to the disaster, academic background, and psychological distress were not performed. Further refinement of the intervention contents was explored based on responses regarding each participant’s impressions of the program in an open-ended format.

## Results

### Participants’ characteristics

Data of five and six kindergarten workers who participated in the intervention and control groups, respectively, were analyzed. Data of seven and seven Media Doctor Japan members who participated in the intervention and control groups, respectively, were analyzed. Demographic characteristics and mental health at baseline were not different between the intervention and control groups in both kindergarten workers and Media Doctor Japan members (Table 1).

**Table 1:** Characteristics of participants

	Kindergarten workers		Media Doctor Japan members	
	Intervention group	Control group	Intervention group	Control group
Age, mean (*SD*)	24.6 (4.5)	31.3 (8.7)	58.1 (11.8)	51.1 (11.4)
Sex, female, *n*	5 (100%)	5 (83%)	3 (43%)	5 (71%)
Relocation, *n*				
Yes, due to the disaster	0 (0%)	0 (0%)	0 (0%)	0 (0%)
Yes, due to other reasons than the disaster	0 (0%)	0 (0%)	0 (0%)	1 (14%)
No	5 (100%)	6 (100%)	7 (100%)	6 (86%)
Educational background, *n*				
Junior college or vocational school	4 (80%)	5 (83%)	0 (0%)	0 (0%)
University or graduate school	1 (20%)	1 (17%)	7 (100%)	7 (100%)
Psychological distress, K6, mean (*SD*)	2.8 (3.1)	3.8 (6.5)	2.7 (3.7)	5.7 (5.3)

*K6* The score of the Kessler 6-item Psychological Distress Scale

### Effectiveness of the contents and delivery methods for the intervention program

**Fig. 2:**
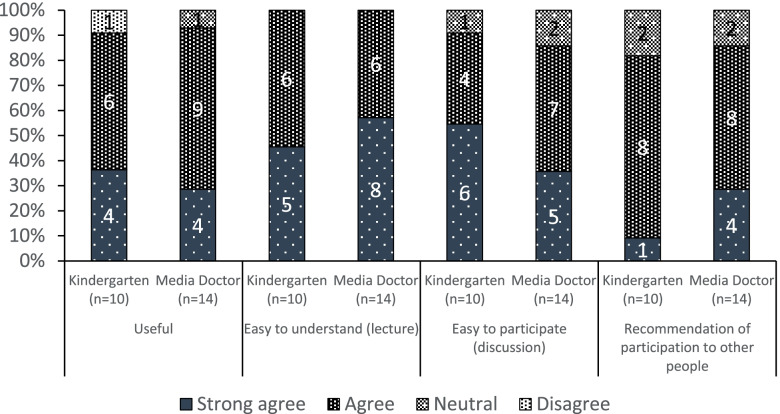
Outcome measures in the intervention and control groups

Participants’ responses to the four questions regarding acceptability are summarized in Fig. 2. One participant from the kindergartens gave a negative response regarding usefulness. Except for this, all participants gave neutral or positive responses. Regarding the remaining three assessment axes of acceptability, every participant from both the kindergartens and Media Doctor Japan gave neutral or positive responses (Fig. 2).

### Appropriateness of potential outcome measures for a future study

Scores of outcome measures from kindergartens and the Media Doctor Japan are summarized separately in Tables 2, 3, and 4. Although the significance was not determined, self-confidence was improved in the intervention group of kindergarten teachers (Table 2). Scores of knowledge regarding radiation health effects were found to be higher after the intervention in both kindergarten teachers and Media Doctor Japan members, and the amount of change was higher in the intervention group than in the control group (Table 3). HL was almost unchanged in the intervention group of participants from both kindergartens and the Media Doctor Japan (Table 4).

**Table 2:** Self-confidence at pre- and post-intervention in the intervention and control groups

	Intervention group			Control group		
	Pre	Post	Change Post-Pre	Pre	Post	Change Post-Pre
Kindergarten	1.2 (0.4)	2.6 (0.5)	1.4 (0.9)	1.8 (0.8)	1.8 (0.8)	0.0 (0.0)
Media Doctor	2.4 (0.8)	2.7 (0.5)	0.3 (0.5)	2.6 (0.8)	2.7 (0.8)	0.1 (0.7)

Variables are presented as mean (*SD*)

**Table 3:** Knowledge regarding radiation at pre- and post-intervention in the intervention and control groups

	Intervention group			Control group		
	Pre	Post	Change Post-Pre	Pre	Post	Change Post-Pre
Kindergarten	2.2 (0.8)	4.6 (0.9)	2.4 (0.5)	1.0 (1.3)	2.0 (2.1)	1.0 (2.0)
Media Doctor	3.0 (1.4)	5.0 (0.0)	2.0 (1.4)	3.4 (1.4)	3.6 (1.6)	0.1 (0.7)

Variables are presented as mean (*SD*)

**Table 4:** Health literacy at pre- and post-intervention in the intervention and control groups

	Intervention group			Control group		
	Pre	Post	Change Post-Pre	Pre	Post	Change Post-Pre
Kindergarten	3.3 (0.5)	3.4 (0.8)	0.1 (0.7)	3.1 (0.5)	2.9 (0.7)	−0.1 (0.4)
Media Doctor	4.2 (0.4)	4.3 (0.4)	0.1 (0.3)	4.1 (0.5)	3.8 (0.6)	−0.2 (0.5)

Variables are presented as mean (*SD*)

### Further refinement of intervention contents

Participants who completed a free-text form generally gave constructive opinions for refinement of the intervention program, for example,

“Each of us has different thoughts and ideas, so it was a good opportunity for us to exchange information and ideas.” (Participant from kindergarten)

In contrast, participants from the Media Doctor Japan raised forthcoming challenges for future iterations of this intervention program. The following are from the participants of the Media Doctor Japan.

#### Regarding program contents

“I think it is also necessary to know when it was written, who wrote it, and for what purpose.”

“I thought it would be good to check what was taught in real-life articles and social networking posts.”

“I think it would be better to narrow down the discussion to a single point before starting.”

#### Regarding setting and management of contents

“It would have been better if there were more participants.”

“If there were more people in the group, there would have been more diverse opinions. I think it would be better to have a team of about five or six people.”

No harmful events occurred or were reported regarding this intervention program.

## Discussion

This study examined the feasibility of a novel program aimed at enhancing self-confidence of kindergarten employees in dealing with radiation-related health concerns from parents. Several previous studies have also focused on enhancing participants’ self-confidence [31–34]. In addition, Orui et al. [35] developed a gatekeeper training program that targeted counselors following the GEJE. However, to the best of our knowledge, there have been no similar studies targeting persons who are not specialized in dealing with anxiety of the public. This study targeted persons who regularly and frequently communicated with parents with young children. Our attempt is expected to deliver the intervention program to broader populations who could benefit from the program.

### Interpretation

#### Recruitment and retention

Of all staff working in the two kindergartens, the proportion of those who participated in the program was 60%. Since all staff were asked to participate in this program on a voluntary basis, this result might be helpful in assuming participation rates in general kindergartens. In preparation for the future intervention, it should be recognized that this intervention program could be delivered to only individuals with relatively high interest in literacy or measure to deal with anxiety of parents with small children. However, there was no serious problem that might affect the generalizability of the results of this study as far as future studies will be conducted in accordance with the procedure described in this paper.

In addition, participants were recruited via representatives of their workplaces. We believe that request for study participation made indirectly through workplace representatives rather than directly by members of our research team may have resulted in less enthusiastic presentation of the program, probably resulting in a decrease in the participation rate. Since the number of staff working at a kindergarten is generally not large, an intervention program involving participants from multiple kindergartens should be considered. Although it is inevitable that participants took some effort to come to the venue, the organizer of this program should consider choosing a venue that is more convenient to access for the intervention program. Furthermore, even though the intervention program was held on weekends, organizers should keep in mind that sudden absence due to work circumstances was possible, thereby resulting in reduced participation rates.

#### Effectiveness of the contents and delivery methods for the intervention program

The results of the acceptability assessment in this study were generally favorable, and the feasibility of the study was thus acceptable. One of the novelties of this study is that the participants were kindergarten teachers, who are often consulted by parents of children. In addition, this study adopted the strategy of enhancing HL, which has been identified as a factor associated with anxiety in previous studies that determined the health effects of radiation. Literacy involves not only obtaining and understanding information but also using it. For this reason, this study is unique in that it incorporates passive lectures and discussions on communicating health information to others.

Several previous studies have used self-confidence as an outcome, especially in Japan, where many studies have been conducted on mothers at the child-rearing age. Kawamata et al. [36] found that the longer length of career as a nursery school or kindergarten teacher was associated with lower reported burnout, and suggested that more experience and increased knowledge about working with young children improved teachers’ self-confidence. Another study by Tomita et al. [37] suggested that daily life activities that improve children’s scientific views and skill encourage educators at nurseries to be confident in implementing science education. The present study differs from previous studies in that it investigated radiation health anxiety and health literacy regarding radiation. These topics are not well known, and participating teachers did not have prior opportunities to learn about them. Therefore, the intervention program is a new attempt to address these lapses in knowledge and is not based on previous studies.

The program was evaluated favorably in terms of ease of participation and encouragement of others to participate, and the feasibility of the program as a whole was judged to be good.

#### Appropriateness of potential outcome measures for a future study

The outcome measures used in this study were intended for those who were currently working in kindergartens, and there were no particular response problems. Furthermore, as mentioned in the previous section, there was an improvement in self-confidence of the kindergarten staff after the intervention, indicating that the results of this study are in the right direction. Although no previous study developed the specific scale which is validated for assessing self-confidence in dealing with radiation-related health concerns, this 4-point Likert scale can be appropriately used based on a previous report suggesting that a Likert scale has been most frequently used to assess self-confidence [38].

Regarding HL, the tool used in this study evaluates HL in general. The present study deals with radiation health concerns, which are different from general health concerns. However, the use of this scale was appropriate because health literacy can be measured at different levels, corresponding to higher level “health literacy” skills that relate to the acquisition, understanding, and application of context-specific knowledge [39].

#### Further refinement of intervention contents

Based on the participants’ comments, several issues were identified. The participants who raised issues were all from the Media Doctor Japan group. They provided many suggestions concerning changes needed to improve the intervention program for the kindergarten staff. Their suggestions were helpful. There were no serious problems with the ease of participation, but several suggestions for improvement were made regarding the program content, program management, and schedule.

First, regarding the content of the program, it was suggested that the content of the program could be improved by providing detailed information about the articles covered (author, date and time of writing, and purpose of writing). In addition, there was an opinion that practice based on actual articles should be implemented. Lastly, the group discussion was started by asking the participants to share their own experiences of what they were worried about during the Great East Japan Earthquake; some suggested that it would be better to specify a single, focused discussion points at the beginning of the group discussion. Since these items are largely up to the program facilitator, it may be effective to adjust these issues if the discussion does not converge well in future studies.

In terms of the program’s management and schedule, there were several comments that it would have been better if the number of participants in the discussions had been larger so that more opinions could have been heard. In some cases, discussions were held among as few as two people plus the facilitator. When the number of participants in the program on a given day was less than five, it would be more meaningful for the participants to have a discussion with about five people in one group, instead of dividing them into two groups. As for the discussion time, some answered that it was just right, while others said they wished they had more time. If the number of participants in the discussion increases, more time may be needed than what was allotted in this study. Thus, more time for discussion should be considered in further studies, depending on the situation.

In addition, the participants from Media Doctor Japan participated in the program online, and there were some challenges specific to the online program such as poor access to the URL to the questionnaire and inability to speak up due to the surrounding environment. Thus, although the use of a teleconference system is beneficial in communicating with persons located far away from one another, certain proficiency in using the system is necessary for the smooth process of the intervention program. Therefore, a teleconference system should be implemented with caution when the target population is not familiar with the system. On the other hand, in the face-to-face program conducted for kindergarten staff in this study, no particular problems were encountered. These findings suggest that the face-to-face format has the advantage in facilitating discussions because it is easier to convey the facial expressions and gestures of the participants to each other. Therefore, the style of the intervention program should be chosen in accordance with the situation, as well as the participants’ skills or locations.

### Limitations

This study had some limitations. Since participants within the same kindergartens were divided into intervention and control groups, there is a possibility that participants assigned to the control group were informed of the content of the intervention program by participants in the intervention group, which might have affected the results of this study. However, intervention contents included an activity which could only be accessed by participation in a live program. Thus, the effect of discussion between groups, if present, was likely sufficiently small.

### Generalizability

The generalizability of this study may be limited. This study was conducted at only 2 kindergartens out of 17 in Fukushima, and the directors of these kindergartens were highly cooperative in participating in this program. Therefore, the effect of the intervention program could be overestimated, and generalizability of this study should be assessed with care.

## Conclusions

This study confirms the feasibility of the intervention program. Future trials should involve a greater number of participants so that participants can engage in active discussion. In addition, the time allotted to conduct lectures and group discussions should be increased in future trials in order to further enhance literacy.

## Data Availability

Data underlying the findings in this study cannot be made publicly available due the nature of ethical approval for the study. Interested researchers may submit requests to the Fukushima Medical University’s Ethics Committee (Contact information: Email: rs@fmu.ac.jp) for access to confidential data.

## Abbreviations

GEJE: Great East Japan Earthquake
HL: Health literacy
CCHL: Communicative and Critical Health Literacy
RCT: Randomized controlled trial
